# Prehospital treatment of severely burned patients: a retrospective analysis of patients admitted to the Berlin burn centre

**DOI:** 10.1186/s13049-024-01239-5

**Published:** 2024-08-14

**Authors:** David Josuttis, Marianne Kruse, Philip Plettig, Ida Katinka Lenz, Denis Gümbel, Bernd Hartmann, Simon Steffan Kuepper, Volker Gebhardt, Marc Dominik Schmittner

**Affiliations:** 1 Department of Anesthesiology, Intensive Care and Pain Medicine, BG Klinikum Unfallkrankenhaus Berlin, Warener Strasse 7, 12683 Berlin, Germany; 2 Department of Trauma and Orthopaedic Surgery, BG Klinikum Unfallkrankenhaus Berlin, Warener Strasse 7, 12683 Berlin, Germany; 3 Department of Trauma, Reconstructive Surgery and Rehabilitation Medicine, University Medicine Greifswald, Fleischmannstraße 8, 17475 Greifswald, Germany; 4 Department of Plastic Surgery, Burn Centre, BG Klinikum Unfallkrankenhaus Berlin, Warener Strasse 7, 12683 Berlin, Germany; 5 Medical Faculty Mannheim of Heidelberg University, Ruprecht-Karls-University Heidelberg, Heidelberg, Germany

**Keywords:** Burn injury, Prehospital emergency care, Emergency Medical Service, Fluid resuscitation, Critical care, Prehospital airway management, Advanced trauma care

## Abstract

**Background:**

Prehospital management of severely burned patients is extremely challenging. It should include adequate analgesia, decision-making on the necessity of prehospital endotracheal intubation and the administration of crystalloid fluids. Guidelines recommend immediate transport to specialised burn centres when certain criteria are met. To date, there is still insufficient knowledge on the characteristics of prehospital emergency treatment. We sought to investigate the current practice and its potential effects on patient outcome.

**Methods:**

We conducted a single centre, retrospective cohort analysis of severely burned patients (total burned surface area > 20%), admitted to the Berlin burn centre between 2014 and 2019. The relevant data was extracted from Emergency Medical Service reports and digital patient charts for exploratory data analysis. Primary outcome was 28-day-mortality.

**Results:**

Ninety patients (male/female 60/30, with a median age of 52 years [interquartile range, IQR 37–63], median total burned surface area 36% [IQR 25–51] and median body mass index 26.56 kg/m^2^ [IQR 22.86–30.86] were included. The median time from trauma to ED arrival was 1 h 45 min; within this time, on average 1961 ml of crystalloid fluid (0.48 ml/kg/%TBSA, IQR 0.32–0.86) was administered. Most patients received opioid-based analgesia. Times from trauma to ED arrival were longer for patients who were intubated. Neither excessive fluid treatment (> 1000 ml/h) nor transport times > 2 h was associated with higher mortality. A total of 31 patients (34,4%) died within the hospital stay. Multivariate regression analysis revealed that non-survival was linked to age > 65 years (odds ratio (OR) 3.5, 95% CI: 1.27–9.66), inhalation injury (OR 3.57, 95% CI: 1.36–9.36), burned surface area > 60% (OR 5.14, 95% CI 1.57–16.84) and prehospital intubation (5.38, 95% CI: 1.92–15.92).

**Conclusion:**

We showed that severely burned patients frequently received excessive fluid administration prehospitally and that this was not associated with more hemodynamic stability or outcome. In our cohort, patients were frequently intubated prehospitally, which was associated with increased mortality rates. Further research and emergency medical staff training should focus on adequate fluid application and cautious decision-making on the risks and benefits of prehospital intubation.

**Trial registration:**

German Clinical Trial Registry (ID: DRKS00033516).

**Supplementary Information:**

The online version contains supplementary material available at 10.1186/s13049-024-01239-5.

## Background

Severe burn injuries are relatively rare, and its incidence in high income countries has been decreasing in recent years [1]. The treatment of these burn patients is exceptionally challenging and requires specialised care in specific burn centres, where sophisticated initial shock management and surgical wound treatment as well as comprehensive multidisciplinary teamwork are needed [2].

While in hospital intensive care treatment is regularly scientifically evaluated and subject to constant improvement, there is still a significant lack of knowledge regarding optimal prehospital care.

The American and German guidelines recommend primary transport of burn patients to specialised centres if certain criteria are met. These include full-thickness (third-degree) burns, an affected body surface area > 10% or suspected inhalation injury among others [3, 4]. The British Guidelines recommend initial transport to local hospitals [5]. There is limited evidence suggesting that patients who require specialised care might benefit from direct transport to burn centres [6].

BG-Klinikum Unfallkrankenhaus Berlin is specialised in emergency and acute care and rehabilitation of seriously ill and severely injured patients. Overall, there are more than 600 beds and 25 departments. It runs one of the 26 German burn centres and is responsible for a large catchment area of more than 30 000 km^2^ with about 7 million inhabitants. Regularly all severe burn cases from Berlin, Brandenburg as well as southern and eastern parts of Mecklenburg-Vorpommern are treated at the centre. It has twelve intensive care beds specifically dedicated and equipped to burn patient care and it treats about 20–30 patients with severe burn of more than 20% TBSA yearly.

Intervals from trauma to ED arrival have been shown to exceed one hour even in urban German regions with dense burn centre coverage [7]. There are no data available for our area regarding the transport times of severely burned patients.

In German EMS paramedics (“Notfallsanitäterin” or “Notfallsanitäter”) are regularly recertified after the primary training of three years and generally allowed and required to administer fluids or drugs including analgesia autonomously according to regional protocols [8]. In addition, there are emergency physicians who are deployed by vehicle or helicopter and supplement the ambulance teams in severe cases. These emergency physicians are required to have at least two years of in-hospital-experience and regionally differing levels of additional training. Over 50% have a background in anesthesiology but also internists, surgeons, internists or neurologists participate in prehospital emergency medicine [9].

In a prehospital setting, clinicians face a variety of challenges when treating burn victims.

After rescue, patients should be examined for potential additional mechanical trauma. Before wound dressings are applied, Emergency Medical Service (EMS) personnel should assess burn size. In recent years, several studies have reported relevant difficulties in the prehospital estimation of total burned surface area (TBSA). In particular, the size of smaller burn wounds (< 20%) seems to be generally overestimated by clinicians who are inexperienced in this regard [10]. However, Maudet et al. recently demonstrated good agreement between the estimations of EMS physicians and the final burn size as diagnosed during assessment in hospital [11].

EMS teams should focus on avoiding hypothermia and the administration of adequate analgesia. The German guidelines suggest the prehospital use of opioids as monotherapy for patients with < 15% TBSA and a combination of intravenous ketamine and midazolam for larger burn injuries [4].

There is an ongoing debate on the indications for intubation in burn trauma patients. General anesthesia is considered an important part of shock treatment because systemic oxygen demand decreases. Additionally, pulmonary gas exchange might be more predictable with patient-adjusted ventilation via a secured airway. Due to the inherent risk of airway swelling after inhalation injury and potentially impaired oxygen delivery or utilization after cyanide or carbon monoxide intoxication, there usually is a low threshold for prophylactic intubation in burn patients. In contrast to these considerations, there is growing evidence in favor of avoiding unnecessary intubations [12, 13].

A further challenging task for EMS teams is targeted fluid administration during early, prehospital resuscitation. There are several formulas for the empirical calculation of fluid needs of severely burned patients, with the most important being Parkland-Baxter and Brooke [14]. While these formula-based approaches are recommended for use during the first day of in-hospital treatment, there is controversy regarding the optimal amount of fluid in the prehospital phase. Because volume overload is a possibly deleterious complication during burn trauma treatment, a cautious administration of fluid is generally recommended.

As previously stated, the reliability of prehospital burn size estimations is questionable, and fluid calculations involving the TBSA could lead to erratic fluid amounts. Several recommendations therefore suggest the initiation of fluid resuscitation with a fixed fluid amount per hour in the prehospital setting [15, 16]. The German guidelines advocate the use of 500–1000 ml of crystalloid during EMS treatment [4].

Currently there is insufficient evidence on the potential association of transport times, prehospital fluid administration or intubation with outcomes of severely burned patients.

In our study we analysed the EMS treatment of severely burned patients in the Berlin metropolitan region. One goal was to outline to what extend German EMS treatment adheres to burn guidelines recommendations. We also aimed to investigate the association of the prehospital treatment characteristics with parameters of hemodynamic stability upon arrival to the emergency department (ED) and 28-day-mortality as primary outcome. Our intention was to contribute to the update of prehospital treatment guidelines, to the development of EMS training concepts and to hypothesis generation for future prospective studies on early treatment of severely burned patients.

## Methods

We conducted a retrospective analysis of patients who were admitted to our burn centre at BG-Klinikum Unfallkrankenhaus Berlin between 2014 and 2019. Patients with flame injuries or scald burns were included. The study was approved by the local Ethics Committee in January 2021 (Eth-44/20), registered at the German Clinical Trial Registry (ID: DRKS00033516) and is presented according to the STROBE guideline [17]. According to federal law, there was no requirement for written patient consent.

**Fig. 1:**
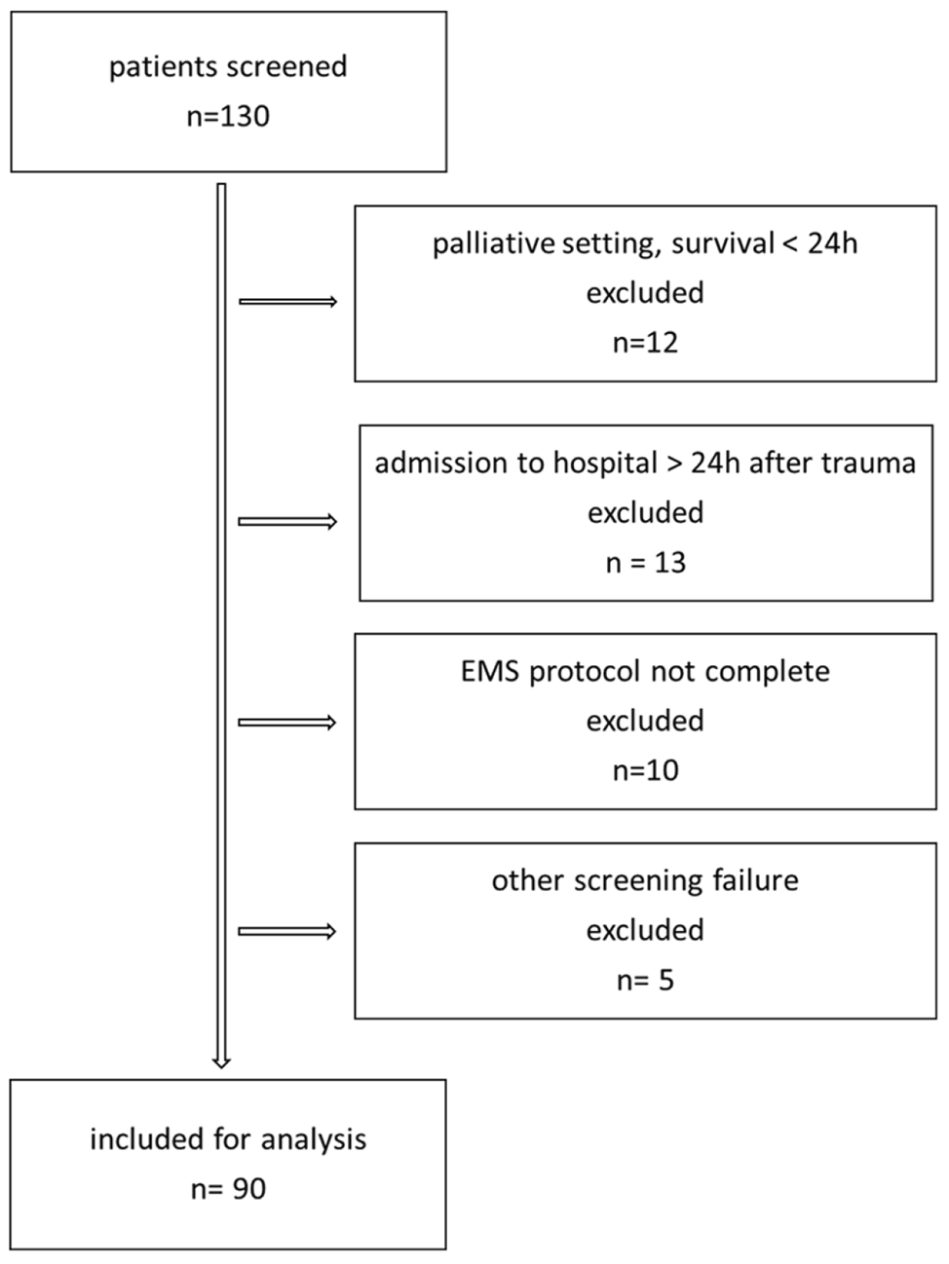
Screening process with in- and exclusion

We included adult patients with > 20% TBSA. Patients who were suffering from toxic epidermal necrolysis were not included. Patients who were treated with palliative care primarily after admission were excluded, as were those who were admitted to the burn centre more than 24 h after trauma (Fig. 1).

Clinical data on patient admission were extracted retrospectively from paper-based patient records and from digital patient intensive care unit (ICU) charts (ICM, Dräger, Luebeck, Germany). Additionally, we used the available data from the hospital data management system (Medico, Cerner Health Services, Berlin, Germany). Baux score was calculated as previously published [18]. Abbreviated Burn Severity Index (ABSI) was also extracted from the clinician’s documentation. Both scores are commonly used to stratify the mortality risk of newly admitted burn patients in ICU. The estimation of TBSA was made at the time of first surgical assessment and debridement. During this procedure bronchoscopy was performed to diagnose or to exclude inhalation injury.

We recorded death within 28 days after admission as primary outcome.

After rigorous plausibility checks, data was extracted in a pseudonymous manner and imported into SPSS (27, IBM, Armonk, NY, USA).

An exploratory data analysis mainly focusing on cohort descriptives was conducted. Unless otherwise indicated, the study parameters are presented as medians with interquartile ranges (IQRs). For group comparisons, the nonparametric Mann‒Whitney U test was performed. Categorical variables are shown as counts and percentages. Frequencies were compared with the chi-square test. A two-sided *P* value < 0.05 was considered to indicate statistical significance. We used the chi-square test for the calculation of odds ratios. To adjust for confounders, we performed bivariate logistic regression analyses.

## Results

We included 90 patients (male/female 60/30, median age 52 [IQR 37–63] years, median TBSA 36% [IQR 25–51], median body mass index 26.56 [IQR22.86-30.86]kg/m^2^). The characteristics of the patient cohort are shown in Table 1.

**Table 1:** Demographic and morphometric variables of the study population and characterization of burn severity

		Total	Mortality		*p*-value
		*n* = 90	survived to hospital discharge *n* = 59	deceased *n* = 31	
Female Sex	Count (%)	30 (33.3%)	16 (27.1%)	14 (45.2%)	0.102
Male Sex	Count (%)	60 (66.7%)	43 (72.9%)	17 (54.8%)	
Age (years)	Median (IQR)	52 (37–63)	48 (34–56)	60 (49–73)	< 0.001
weight (kg)	Median (IQR)	85 (68–95)	85 (65–95)	85 (69–100)	0.537
BMI (kg/m^2)	Median (IQR)	26.56 (22.86–30.86)	26.58 (22.86–30.67)	26.04 (22.86–31.49)	0.859
TBSA (%)	Median (IQR)	36 (25–51)	35 (25–50)	43 (29–65)	0.076
Inhalation Injury	Count (%)	46 (52.9%)	25 (43.1%)	21 (72.4%)	0.012
Full thickness burn	Count (%)	77 (85.6%)	47 (79.7%)	30 (96.8%)	0.003
ABSI-Score	Median (IQR)	9 (8–11)	8 (7–10)	11 (9–13)	< 0.001
Baux-Score	Median (IQR)	104 (86–120)	94 (78–108)	121 (106–130)	< 0.001

A total of 31 (34.4%) patients died within a median of 13 (IQR 5–32) days after admission. Among the 59 surviving patients, the median duration of intensive care treatment was 12 (IQR 5–32) days.

As shown in Table 1, the patients who survived to hospital discharge were younger and had lower ABSI (8 vs. 11, *p* < 0,001) and Baux scores (94 vs. 121, *p* < 0,001). Although the frequencies of full thickness burns (“third degree”) and inhalation injury were significantly greater in the group of deceased patients, the trend toward a larger burns in this group did not reach statistical significance.

The mean time from trauma to ED arrival was 1 h and 45 min; within this time, an average of 1961 ml of crystalloid fluid was administered (median 0.48 ml/kg/% TBSA, IQR 0.32–0.86, Table 2).

**Table 2:** Prehospital treatment and vital signs upon arrival to emergency department (ED) survivors and non-survivors

		Total	Mortality		*p*-value
			Survived to hospital discharge *n* = 59	deceased *n* = 31	
Time from trauma to ED arrival (min)	Median (IQR)	105 (73–140)	105 (73–141)	110 (71–128)	0.875
Total prehospital fluid administration (ml)	Mean (Standard deviation)	1961 (1603)	1887 (1519)	2100 (1770)	0.833
Prehospital Fluid administration per hour (ml/h)	Median (IQR)	892 (601–1286)	891 (571–1333)	923 (670–1250)	0.665
Prehospital fluid administration per %BSA and weight (ml/%BSA/kg)	Median (IQR)	0.48 (0.32–0.86)	0.49 (0.34–0.92)	0.36 (0.22–0.75)	0.231
Vasopressor administration upon arrival to ED	Count (%)	8 (8.9%)	3 (5.1%)	5 (23.4%)	0.118
Prehospital analgesia with ketamine	Count (%)	27 (30%)	18 (30.5%)	9 (29.0%)	1
Prehospital analgesia with opioid	Count (%)	67 (74.4%)	42 (71.2%)	25 (80.6%)	0.447
Prehospital intubation	Count (%)	55 (61.1%)	29 (49.2%)	26 (83.9%)	0.001
If ventilated: Fraction of inspired oxygen upon arrival to ED	Median (IQR)	0.8 (0.5-1)	0.6 (0.5-1)	0.8 (0.7-1)	0.045
If spontaneously breathing: Oxygen administration upon arrival to ED (l/min)	Median (IQR)	6 (4–6)	4 (4–6)	8 (3–10)	0.383
Heart rate upon arrival to ED (1/min)	Median (IQR)	92 (84–112)	93 (86–108)	91 (80–118)	0.896
Mean arterial pressure upon arrival to ED (mmHg)	Median (IQR)	93 (80–106)	93 (80–110)	86 (63–98)	0.022
SpO2 upon arrival to ED (%)	Median (IQR)	98 (96–100)	98 (96–100)	98 (96–99)	0.555
Temperature upon arrival to ED (°C)	Median (IQR)	35.9 (35.3–36.8	35.9 (35.3–36.8)	35.8 (35.2–36.8)	0.94
pH upon arrival to ED	Median (IQR)	7.32 (7.26–7.39)	7.32 (7,27 − 7,39)	7,32 (7.19–7.38)	0.427
Lactate upon arrival to ED (mmol/l)	Median (IQR)	2 (1.5-3)	1.7 (1.4–2.7)	2.5 (1.6–4.6)	0.027
Base excess upon arrival to ED	Median (IQR)	-3.3 ((-7.4)-(-1.2))	-3.2 ((-6.1)- (-1.1))	-4.8 ((-8.5)-(-1.2))	0.213

**Fig. 2:**
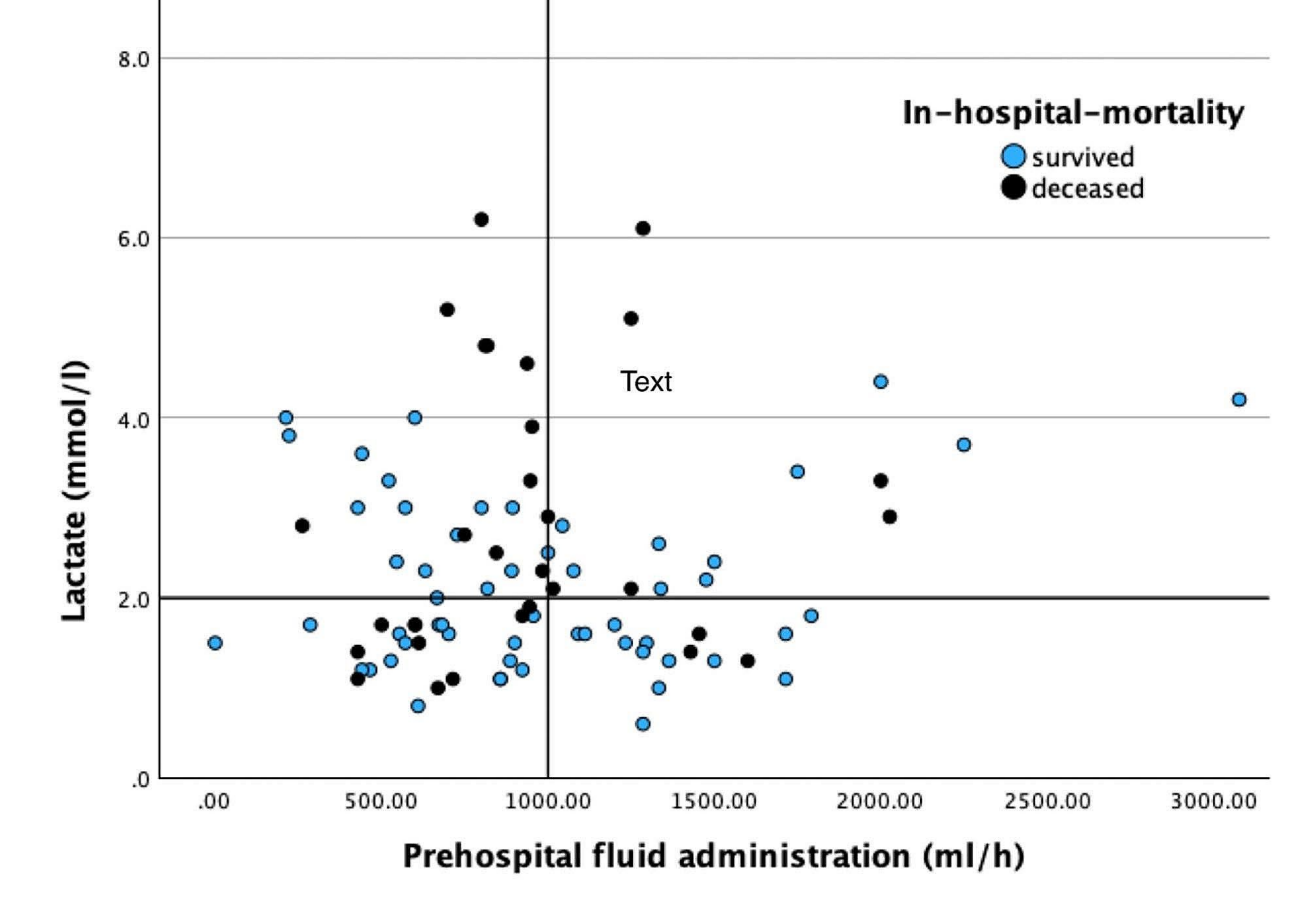
Lactate levels upon arrival in ED (in mmol/l) in relation to the amount administered fluids per prehospital hour (in ml/h), *p* = 0,741 (linear regression)

Two patients (2.2%) arrived without documented EMS fluid administration. A total of 67.7% of the patients received more than 1000 ml before arrival at the emergency department, 34 (37.7%) patients received more than 1000 ml per hour. On arrival, 12.2% (*n* = 11) of patients were hypotensive, and 50% had a lactate greater than 2 mmol/l. There was no association between fluid administration and lactate upon ED arrival according to linear regression analysis (*p* = 0.741, Fig. 2).

Considering analgesia, 30% of patients received ketamine, and 74.4% of patients were given opioids. In 15 patients, a combination of both agents was administered. In 11 patients (12,2%), there was no documentation of any analgesia administered by EMS personnel.

A lower proportion of survivors underwent prehospital intubation (49.2% vs. 83.9%, *p* = 0.001). Among those patients, survivors were ventilated with lower FiO_2_ (0.6 vs. 0.8, *p* = 0.045). Patients who were intubated by EMS personnel had a longer prehospital treatment interval (115 vs. 90 min, *p* = 0.045). The rate of inhalation injury and TBSA were greater in the group that was intubated (spontaneous breathing vs. intubation 30% vs. 45% TBSA, *p* < 0.001 and 37.1% vs. 62.3% occurrence of inhalation injury, *p* = 0.029), as depicted in additional Table S1.

**Fig. 3:**
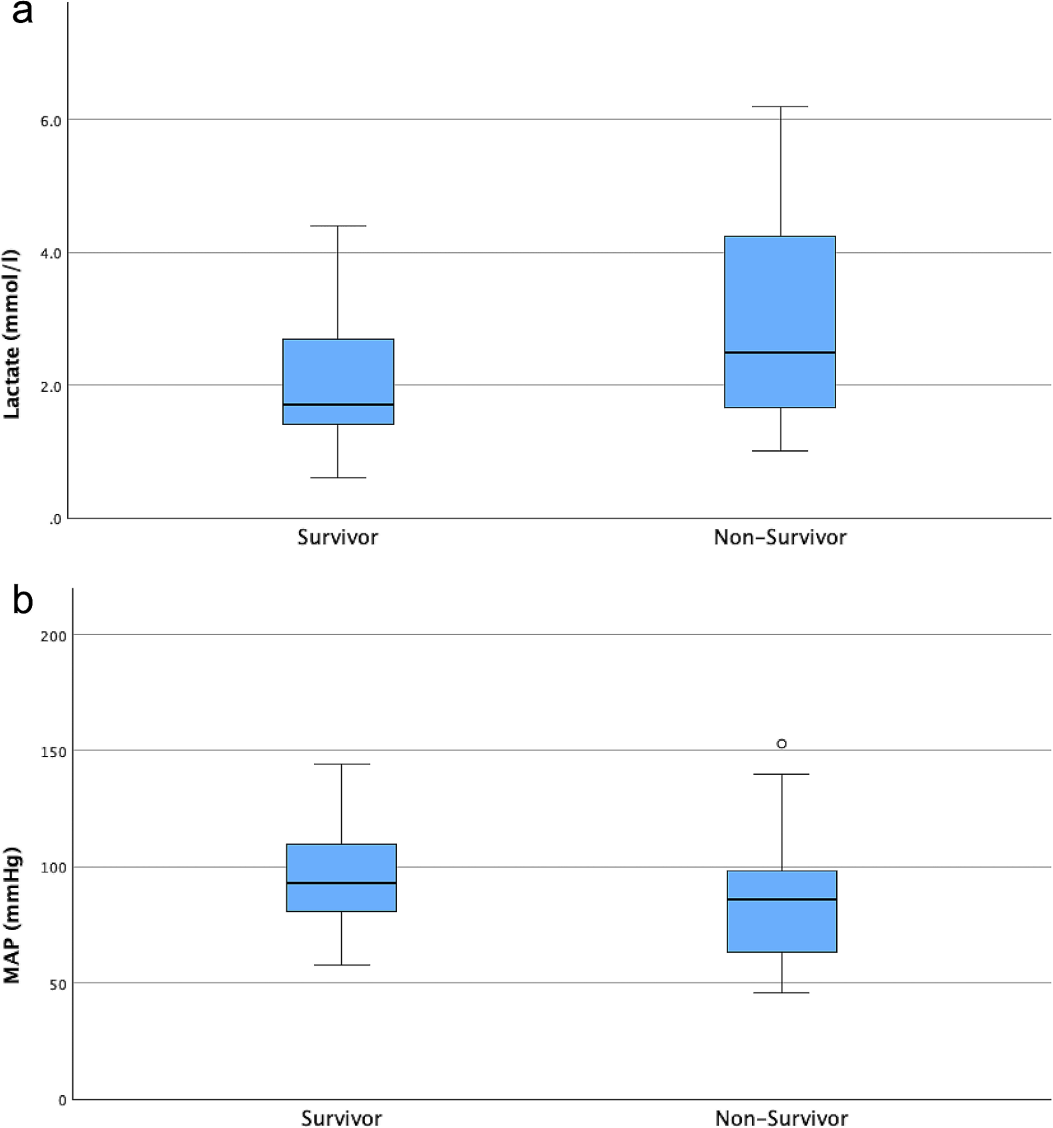
(**A**) Lactate levels upon arrival to ED (mmol/l) comparing survivors and non-survivors, *p* = 0,027. (**B**) Mean arterial pressure (MAP, in mmHg) upon arrival to ED comparing survivors and non-survivors, *p* = 0,022

Nonsurvivors had higher lactate levels (2.5 vs. 1.7 mmol/l, *p* = 0.027; Fig. 3a) and lower blood pressures (mean arterial pressure 86 vs. 93 mmHg, *p* = 0.022; Fig. 3b) at the time of arrival to the hospital. Neither the amount of fluid administered, nor the frequency of prehospital vasopressor use was significantly different between survivors and patients who died later.

We divided the cohort into patients who received up to 1000 ml/h fluid before arrival at the emergency department and those who were given more fluid. In the group of patients who received excessive fluid, the time from trauma to arrival was shorter (80 vs. 115 min, *p* = 0.004, Table 3).

**Table 3:** Group comparison regarding patient characteristics and prehospital treatment between patients who received 0-1000ml per prehospital treatment hour vs. those who received more

		Prehospital fluid administration in ml/h		*p*-value
		0-1000 *n* = 56	> 1000 *n* = 34	
Age (years)	Median (IQR)	55 (40–67)	50 (36–56)	0.153
BMI (kg/m^2)	Median	26.59 (23.88–31.04)	26.56 (22.24–29.35)	0.278
TBSA (%)	Median	36 (29–51)	37 (25–60)	0.997
inhalation injury	Count (%)	28 (50%)	18 (56.3%)	0.659
full-thickness burn	Count (%)	51 (91.1%)	26 (76.5%)	0.069
Time from trauma to ED arrival (min)	Median (IQR)	115 (86–168)	80 (70–112)	0.004
Prehospital fluid administration per %BSA and weight (ml/%BSA/kg)	Median (IQR)	0.37 (0.23–0.62)	0.83 (0.47–1.1)	< 0.001
Prehospital analgesia with opioid	Count (%)	39 (69.6%)	28 (82.4%)	0.219
Prehospital analgesia with ketamine	Count Column *N* %	14 (25.0%)	13 (38.2%)	0.237

**Fig. 4:**
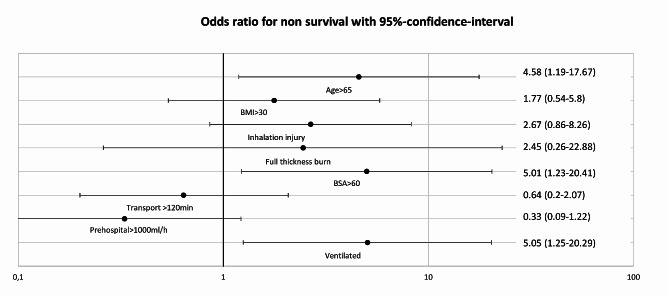
Odds ratio for death in multivariate regression analysis correcting for given confounders. Values are given with 95%-confidence-interval (error bars). BMI: Body mass index, BSA: body surface area Parameters of model fitness are p = 0,002, Nagelkerkes R-Square = 0,41 (mortality), *p* < 0.001. X-axis in logarithmic scale

The results of multivariate regression analysis controlling for known confounders influencing patient outcomes after severe burns (age > 65 years, BMI > 30 kg/m^2^, inhalation injury, full thickness burn and TBSA > 60%) are shown in Fig. 4. Invasive ventilation at the time of arrival to the hospital was independently associated with increased mortality (multivariate odds ratio 5.05 (95% CI: 1.25–20.29)).

## Discussion

In this retrospective analysis of severely burned patients, we found that patients received 0.48 ml/kg/% TBSA of fluids by the EMS team and arrived at the burn centre on average more than 100 min after the injury. Neither the amount of administered crystalloids nor the transport time was associated with the frequency of death. Most patients received opioids as analgesic agents. Intubation was performed prehospitally in more than 61% of the patients, and was associated with higher inhalation injury, larger TBSA and a longer time from trauma to ED arrival. Multivariate regression revealed a significant association between prehospital intubation and mortality.

As previously reported, burn depth and the frequency of inhalation injury as well as predictive score values (ABSI and Baux) are significantly different between survivors and patients who later die. In contrast to other studies, in our cohort, the difference in TBSA between survivors and deceased patients was not statistically significant. This is likely due to our small sample size, and one must consider that extremely severe burns of > 60% TBSA, which are associated with a marked increase in mortality, are generally rare.

Regarding epidemiological variables, our cohort seems to be comparable to larger registry analyses from Australia and New Zealand. Additionally, the extent and severity of burn injuries in industrial countries seem to be similar [19]. Compared to a systematic review from 2010 incidence of severe burns appears to be lower, most likely mainly caused by stricter inclusion criteria in our study [20].

Because of the larger catchment area, our centre receives cases with longer transport intervals compared to the previously published data from the Cologne area, which has a denser network of several burn centres [7]. Interestingly, despite longer prehospital treatment, most patients show relatively stable vital signs upon arrival to ED and rarely experience hypotension, tachycardia or hypoxia on admission to the emergency department. In our study, longer transport times were not associated with higher mortality rate.

Prehospital treatment often diverges from national recommendations. Analgesia is only briefly covered in the German guidelines, but the suggestion focuses on ketamine and midazolam for the patient group represented in our cohort. EMS personnel seemed to choose a more nuanced approach, and most patients received opioids, especially if they were intubated. Considering that more than 17% of patients were given a combination of ketamine and opioids, the choice of analgesics might reflect individual and personalised decision making. As Maudet et al. demonstrated, opioids seem to be the preferred analgesics in Central Europe [11]. Consistent with previous findings, some patients do not require prehospital analgesia, but their proportion seems to be lower than that in comparable Finnish and French cohorts [11, 21].

A large body of evidence emphasizes the importance of cautious prehospital fluid administration [4, 15, 16]. Even the most liberal interpretation of the existing guidelines, recommending a maximum of 1000 ml per hour, was exceeded in more than 37% of patients in our cohort. Although greater awareness of current treatment goals during emergency care is desirable, we found no significant association between excessive fluid intake and measured outcome parameters. Patients who received excessive amounts of fluids had shorter transport intervals. Most likely, in the task-loaded and stressful initial treatment phase, the emphasis is on timely initiation of fluid administration, and longer en-route intervals provide the opportunity to reevaluate, look up standard operating procedures and adjust infusion drips.

There was no association between fluid administration and vital signs at arrival. In particular, the potential for improved microcirculation following excessive fluid administration directly after burn injury is not supported by our data, as there is no association with lactate levels upon arrival to ED. Fortunately, excessive fluid application also did not result in increased mortality.

Badulak et al. retrospectively identified risk factors, for long-term intubation in burn patients. These include full-thickness facial burns, stridor, respiratory distress, swelling on laryngoscopy, altered mentation, hypoxia/hypercarbia, hemodynamic instability, suspected smoke inhalation, and singed facial hair or suspected smoke inhalation. Patients displaying these “Denver criteria” should be considered for intubation, whereas patients lacking these features should not be intubated [22]. Evaluation according to the Badulaks recommendation is difficult and not completely feasible in prehospital settings, as some criteria (such as hypercarbia or swelling on laryngoscopy) usually cannot be monitored with the same accuracy by EMS means.

As our cohort consisted of only severely burned patients, a large proportion of patients suffered from inhalation injury, and more than 60% of patients received prehospital intubation. This was associated with longer times from trauma to hospital arrival, which is consistent with observations from the Cologne Burn Centre [23]. Longer prehospital transport times might have influenced the decision to intubate the patient. Several studies have concluded that unnecessary intubations in severely burned patients are common [12, 13], and our findings of increased mortality in ventilated patients mandates diligent consideration before deciding on intubation prehospitally. There have been efforts to identify risk factors for long term intubation in burn trauma patients [22]. Evaluation according to these recommendations usually is not feasible in prehospital settings and the decision to initiate general anesthesia and invasive ventilation in severely burned patients currently heavily relies on clinical judgement.

### Limitations

One major limitation is the retrospective nature of our study. Regrettably, we were unable to analyse the reliability of early burn size estimation due to insufficient EMS documentation. Even though rigorous plausibility checks were carried out, faulty documentation and data extraction are potential sources of shortcomings.

The sample size is limited due to the low incidence of the examined population. This limits the validity of statistical testing, especially as exploratory data analysis is susceptible to alpha-error accumulation. We therefore sought to limit research questions and need to interpret the results with caution.

## Conclusion

In the Berlin metropolitan area, paramedics and emergency physicians caring for severely burned patients need to consider long transport times. Current adherence to prehospital treatment recommendations is unsatisfactory. Because there is no convincing evidence regarding the suggestion to primarily use ketamine in severely burned patients and because Central European practitioners seem to prefer opioids as first-line analgesia, some aspects of the existing guidelines might need to be reviewed. Nevertheless, the international guideline recommendation to limit prehospital fluid administration to fixed amounts is based on reasonable considerations and currently insufficiently executed. The training of EMS staff and emergency physicians should focus on adequate but cautious fluid administration in early resuscitation.

Currently, many patients are intubated after severe burn injuries. Our data underscore the potential for harm from unnecessary intubation. Further research is needed to stratify the indications for prehospital intubation in severely burned patients.

### Electronic Supplementary Material

Below is the link to the electronic supplementary material.

Supplementary Material 1

## Data Availability

The datasets analysed during the current study are available from the corresponding author on reasonable request.

## Abbreviations

ABSI: Abbreviated Burn Severity Index
CI: Confidence interval
ED: Emergency department
EMS: Emergency medical service
ICU: (Intensive care unit)
IQR: Interquartile range
OR: Odds ratio
TBSA: Total body surface area
