# Supplementary material for: Prehospital treatment of severely burned patients: a retrospective analysis of patients admitted to the Berlin burn centre
Source: Scand J Trauma Resusc Emerg Med. 2024 Aug 14;32:70. doi: 10.1186/s13049-024-01239-5 (PMC11323598; doi:10.1186/s13049-024-01239-5)
Supplement: Supplementary file 1 — Supplementary Material 1 [file 13049_2024_1239_MOESM1_ESM.docx]

Additional Table 1

|  | | Prehospital fluid administration in ml/h | | p | Ventilated | | P |
| --- | --- | --- | --- | --- | --- | --- | --- |
|  |  | 0-1000  n=56 | >1000  n=34 |  | not ventilated  n=35 | Ventilated  n=55 |  |
| Hypertension | Count (%) | 13 (23.2%) | 8 (23.5%) | 1 | 13 (37.1%) | 8 (14.5%) | 0.021 |
| Coronary artery disease | Count (%) | 5 (8.9%) | 1 (2.9%) | 0.403 | 3 (8.6%) | 3 (5.5%) | 0.674 |
| Chronic heart failure | Count (%) | 5 (8.9%) | 1 (2.9%) | 0.403 | 3 (8.6%) | 3 (5.5%) | 0.674 |
| Diabetes | Count (%) | 7 (12.5%) | 3 (8.8%) | 0.737 | 7 (20%) | 3 (5.5.%) | 0.043 |
| Pulmonary disease | Count (%) | 1 (1.8%) | 2 (5.9%) | 0.554 | 0 | 3 (5.5%) | 0.279 |
| Liver disease | Count (%) | 5 (8.9%) | 2 (5.9%) | 0.706 | 2 (5.7%) | 5 (9.11%) | 0.701 |
| Neurological/ psychiatric disorder | Count (%) | 13 (23.2%) | 8 (23.5%) | 1 | 8 (22.9%) | 13 (23.6%) | 1 |
| Alcohol abuse | Count (%) | 10 (17.9%) | 3 (8.8%) | 0.356 | 6 (17.1%) | 7 (12.7%) | 0.557 |
| Drug abuse | Count (%) | 3 (5.4%) | 0 | 0.287 | 0 | 3 (5.5%) | 0.279 |

Preexisting medical conditions within subgroups of the study cohort
